# Stem subsidence in total hip arthroplasty: retrospective investigation of a short stem using a simple measurement approach

**DOI:** 10.1007/s00402-024-05642-6

**Published:** 2024-12-16

**Authors:** Nicolas Horst, Christoph Theil, Georg Gosheger, Tobias Kalisch, Burkhard Moellenbeck

**Affiliations:** Department of General Orthopaedics and Tumour Orthopedics, Muenster University Hospital, Albert-Schweitzer-Campus 1, 48149 Muenster, Germany

**Keywords:** Total hip arthroplasty, Short stem, Subsidence Index, Cementless

## Abstract

**Background:**

Uncemented total hip arthroplasty (THA) is a successful treatment for advanced hip joint diseases. More recently, short stems became increasingly popular, but stem subsidence remains a concern. This study investigates early short stem subsidence in a large patient cohort using a simple measurement approach for everyday practice.

**Methods:**

This retrospective, single center, single implant design study included 1000 patients with primary THA. Subsidence was evaluated using standardized weight-bearing radiographs taken 3–5 days and 2–3 weeks postoperatively with full weight-bearing (FWB). A novel Subsidence Index (SID) was introduced to quantify stem subsidence in a simple and reproducible manner. The SID is calculated by averaging four distinct linear measurements between defined anatomical landmarks on the femur and the implant, captured on standard radiographs without additional software.

**Results:**

Out of all analyzed patients 6% (60/1000) had subsidence of more than 3 mm. The mean subsidence was 1.3 mm (range, 0 to 16.25 mm).

There were 0.6% (6) who underwent stem revision for symptomatic subsidence. Men and obese patients had greater subsidence. However, patient age, BMI, stems without lateral bone contact and other demographic factors were not associated with subsidence.

**Conclusion:**

Early subsidence is relatively frequent with this uncemented short stem, however revisions are rare. Patients with risk factors should be counseled regarding FWB and radiographic controls should be performed. The SID provides an easy, non-invasive and inexpensive tool for early subsidence assessment; however, its simplicity limits its accuracy. Further research is needed in comparison to more elaborate methods.

## Background

While historically, uncemented stem fixation was a major critical issue and long-porous coated stems were used to achieve adequate stability, optimized materials and fixation strategies allow for shorter implants that preserve bone stock [1].

One of the most common mechanical phenomena is stem migration of an uncemented stem [2–4]. A certain amount of subsidence is usually detected within the first few weeks or months postoperatively and is considered physiological [5]. However, excessive subsidence beyond this initial period is a cause for concern, as it may compromise the stability of the implant and lead to loosening, instability or fracture [6]. Distinguishing between this physiological subsidence and pathological subsidence is crucial for orthopaedic surgeons for the detection of implant failure. [4, 7–10].

The subsidence of short stems is a well-known and widely discussed, but it has mostly been analyzed in small and specific populations that exclude common patient groups like patients over 70 years or patients with specific risk factors [10–12].

Most studies analyzed the migration pattern with a method called radio stereo-photogrammetric analysis (RSA) or a method called Single-Image-Radiograph-Analysis (EBRA-FCA). However, the RSA method is very expensive and requires an extensive technical setup but is significantly more accurate than the EBRA-FCA method [10].

The primary aim of this study was to investigate the early migration of an uncemented short stem within the first three weeks post-surgery under full weight-bearing conditions by employing conventional anterior–posterior standing pelvis radiographs. Specifically, the study aimed to develop a simple, clinical method for early detection of stem subsidence and to identify patient factors that potentially influence the degree of subsidence. The study was guided by the hypotheses that the Subsidence Index (SID) would provide a reliable and straightforward method for detecting early subsidence of uncemented short stems in THA, and that certain patient demographics and characteristics, such as gender, BMI, and stem contact with lateral cortical bone, would significantly influence the extent of early subsidence. Furthermore, the SID provides a simple tool for communication, for example between orthopaedic surgeons and doctors at the rehabilitation facility.

## Materials and methods

This retrospective study included a group of 1000 Patients who underwent THA surgery at a single high volume orthopaedic specialty hospital.

The study population was selected based on the following inclusion criteria:

- Patients who underwent THA surgery between 2017–2021 using an antero-lateral minimally invasive approach, were treated using a single design short stem (A2, Artiqo, Luedinghausen, Germany)
- Patients were instructed to bear full weight on the operated leg right after surgery and
- Patients had two standardized weight bearing radiographs available for analysis. The first radiograph was taken 3–5 days after the surgery full weight-bearing (due to physiological subsidence after full weight-bearing [4]) and the second one 2–3 weeks after the patients underwent physiotherapy in the hospital’s rehabilitation center.

All radiographs were standardized/calibrated using a 3 cm measuring sphere, consistently performed by the same team of five medical radiology technologists, always in a standing position, and both pre- and postoperative, with identical parameters.

**Fig. 1:**
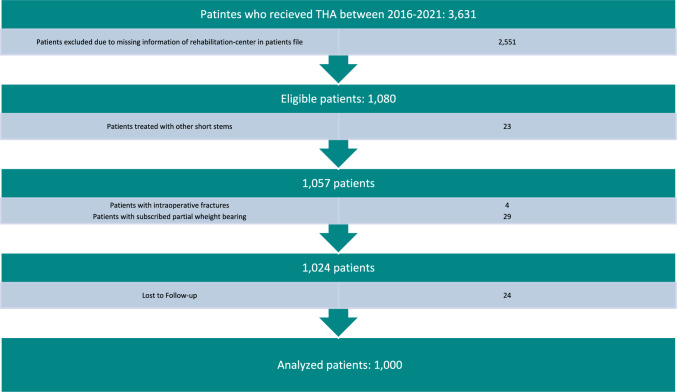
Strobe diagram showing inclusion and exclusion of patients

The study excluded patients (Fig. 1), who

- were recommended partial weight bearing postoperatively due to intraoperative complications or diminished bone quality, (29 Patients)
- had experienced intraoperative complications such as fissures or fractures, (4 Patients)
- received postoperative rehabilitation at another facility or no facility was mentioned, (2551 Patients)
- had missing, low-quality or non-standardized radiographs that were not suitable for analysis, (24 Patients)
- were treated using other surgical approaches or short stems. (23 Patients)

The patients analyzed were randomized as they were admitted to the adjacent rehabilitation center depending on short term availability and patient preference. All patients who underwent postoperative rehabilitation at another facility did not undergo the same standardized radiographic control after 2–3 weeks. This was used as a parameter to identify the study cohort from the hospital’s electronic patient database.

For all patients included in the study, the following data were extracted and analyzed (Table 1):

- age,
- gender,
- weight and BMI at the time of surgery,
- comorbidities with a specific focus on diabetes and/or a history of nicotine and alcohol abuse,
- affected side,
- date of surgery,
- date of the first and second radiograph,
- contact between the short stem and the lateral cortical bone, (Fig. 3)
- specific measurement values obtained from the analyzed images, and the resulting differences.
- possible revision surgery (defined as reoperation where the stem implant or part of the short stem is changed until the end of 2022) with a follow up of 1–5 years (mean 3,1 years)

**Table 1:** Characteristics of this study population divided into age groups * Body mass index, ** Subsidence Index

Characteristic	≤ 75a	> 75a	Total
Men	354 (35.4%)	64 (6.4%)	418 (41.8%)
Women	458 (45.8%)	124 (12.4%)	582 (58.2%)
∅ Weight	82.1 kg	73.2 kg	80.4 kg
∅ BMI*	27.2 kg/m^2^	25.5 kg/m^2^	26.9 kg/m^2^
∅ SID**	1.3 mm (SD = 1.4 mm)	1.3 mm (SD = 1.5 mm)	1.3 mm (SD = 1.4 mm)
Alcoholics	9 (0.9%)	1 (0.1%)	10 (1.0%)
Smoking status	81 (8.1%)	8 (0.8%)	89 (8.9%)
Diabetes	40 (4.0%)	11 (1.1%)	51 (5.1%)
Contact between short stem and lateral bone	426 (42.6%)	103 (10.3%)	529 (52.9%)
Revision	8 (0.8%)	2 (0.2%)	10 (1.0%)
THA on the Right	434 (43.4%)	95 (9.5%)	529 (52.9%)
THA on the Left	378 (37.8%)	93 (9.3%)	471 (47.1%)
∅ Time between radiographs			16.6 days (range, 7 to 43 days)
Total	812 (81.2%)	188 (18.8%)	1000 (100%)

The primary endpoint of the study was the measurement of early stem subsidence in uncemented total hip arthroplasty using the Subsidence Index (SID). Subsidence greater than 3 mm within the first three weeks post-surgery was considered clinically significant.

Secondary endpoints were the identification of patient demographics and characteristics, such as age, gender, BMI, and body weight, that influence the extent of early subsidence as well as the possible influence on whether the patient requires revision surgery.

All Patients underwent the THA surgery due to Osteoarthritis (94.9%), femoral head necrosis (3.8%) or fractures (1.3%).

**Fig. 2:**
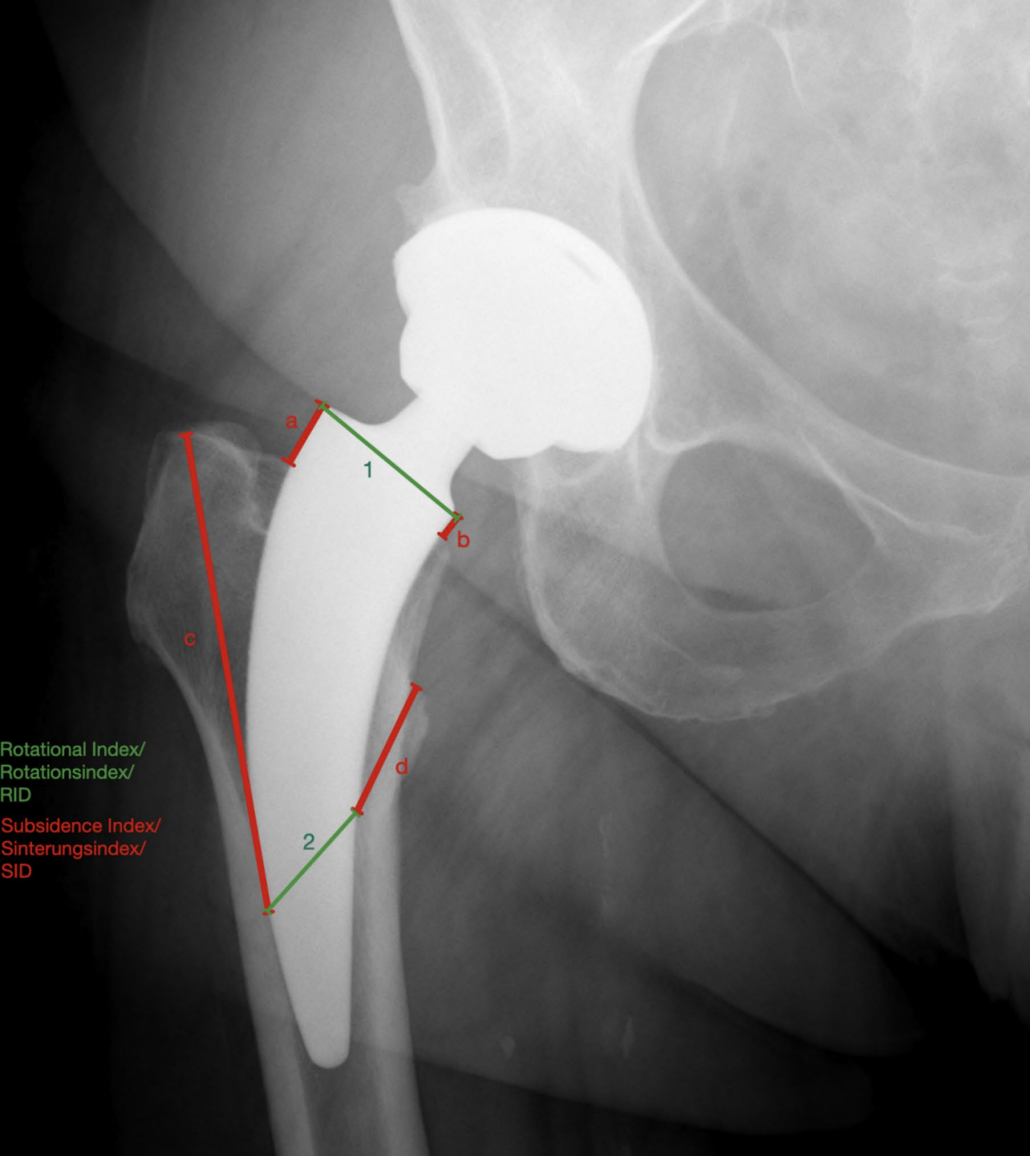
Example of Subsidence- and Rotational-Index shown on conventional standing pelvic radiograph; **a** upper corner of the short stem just below the artificial femoral neck and ending at the point of entry of the short stem into the femur; **b** same distance as (**a**) but on the opposite side of the short stem; **c** distinct mark on the lateral side of the short stem, which is defined by a change in coating, to a specific point above the trochanter major; **d** distinct mark which is defined by a change in coating but on the medial side of the short stem to a specific point at the trochanter minor; (1) diameter just below the artificial femoral neck; (2) medial mark to the lateral mark on the short stem

**Fig. 3:**
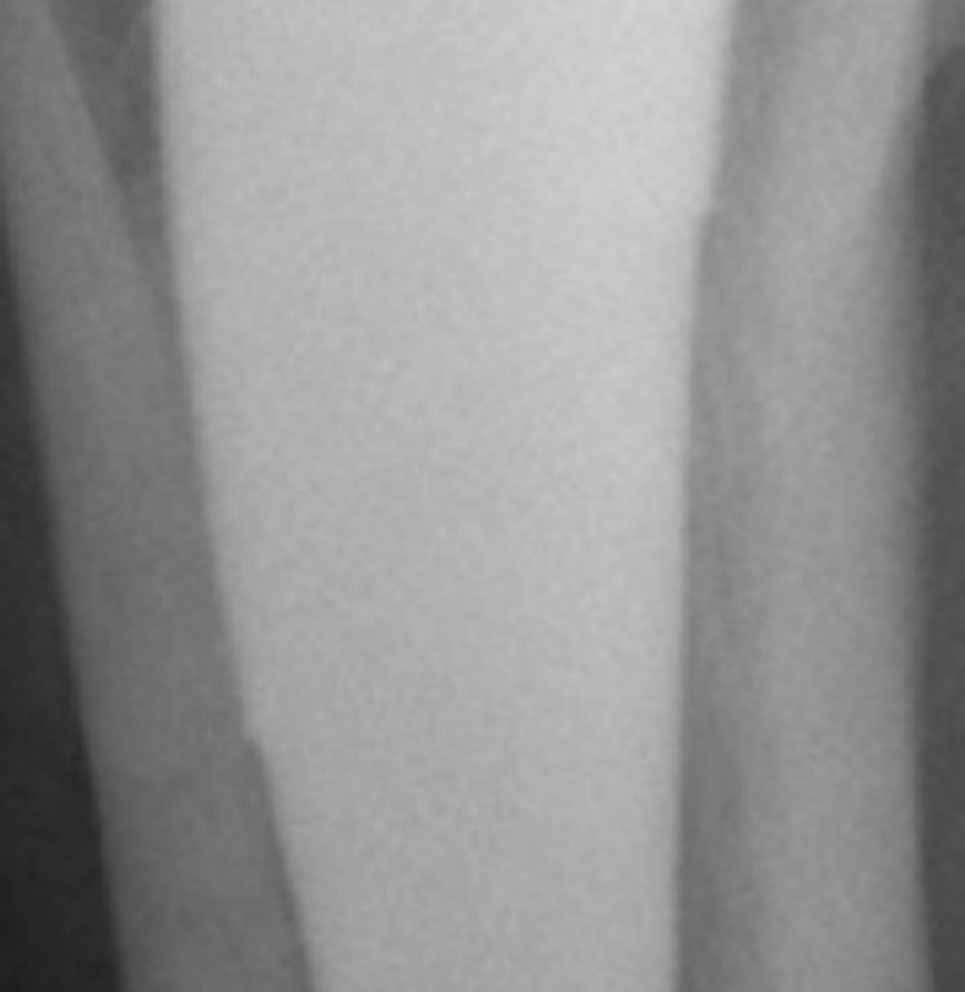
Distinct marking on the short stem caused by the change in coating as well as a short stem with lateral bone contact

The radiographs were analyzed using the Subsidence Index (SID) (a term introduced by the author) a measuring method for an alteration of the position of the short stem inside the femur. For this purpose, 4 distances were measured, all starting at a distinct point on the short stem and ending at a certain point of the femur. If a short stem lacks distinct reference points along its shaft, the tip of the stem may be utilized as an alternative reference. However, ideally stems with partial coating that is visible on radiographs are suitable for the application of the SID. All distances were documented on the first weight bearing radiograph 3–5 days post-surgery and on the second one in an identical fashion. All four values were subtracted from each other forming an absolute value. That value was divided by 4 resulting in the SID. (Figs. 2, 3).

The mean of the distances |(a2-a1)| + |(b2-b1)| + |(c2-c1)| + |(d2-d1)|:4 was then defined as the SID.

Additionally, 2 measurements were used to define the rotational index (RID). The mean of the distances 1 and 2 was then defined as the RID. (Figs. 2, 3) All distances were measured in millimeter using a digital viewer system (Xero Universal Viewer, Agfa Healthcare, Bonn, Germany). To assure the SIDs reliability, prior to the study, 50 random patients were chosen to assess inter- and intra-observer reliability.

Subsidence was defined as every movement of the short stem inside the femur post-implantation. Any subsidence of more than SID > 3 mm was considered clinically relevant. This cut-off value was chosen as the difference between head lengths is > 3 mm for this system across different head diameters.

### Statistical analysis

Descriptive statistics were used to determine data normality. The non-parametric Mann–Whitney U test was used to analyze non-parametric variables. Categorical variables were compared using Fisher’s exact test. The Bonferroni correction was used to adjust p-values accordingly (α = 0,05/7≈0,007). Intraclass Correlation Coefficient was used to assess the inter- and intra-observer reliability. The statistical analysis was done with IMB SPSS® and Microsoft Excel®.

## Results

Out of the 1000 short stems implanted, 6% (60) demonstrated subsidence above the defined cut-off value of SID 3 mm with immediate postoperative full weight-bearing. Among these, 93% (56) had a subsidence between 3 and 10 mm SID, while only 7% (4) had a subsidence above 10 mm SID with a maximum value of 16.25 mm SID. The mean subsidence was 1.3 mm (range, 0 to 16.25 mm).

Out of all analyzed short stems 53% (529) had radiographic contact with the lateral cortex of the bone.

Revision due to complications was necessary in 1% (10) of the cases. Complications were loosening of the stem without any subsidence (0.3%, 3), signs of an infection without any subsidence (0.1%, 1) and pain upon weight-bearing and subsidence (0.6%, 6). Among those, 83% (5) had a SID > 3 mm before being discharged. The one remaining patient did not show any signs of subsidence within the first three weeks.

Patients with a BMI ≥ 30 had a higher, but not significant subsidence than patients with a BMI < 30 (1.48 vs. 1.24 mm, p = 0.011). Overweight patients with a bodyweight ≥ 100 kg on the other hand showed a higher subsidence (1.60 vs. 1.25 mm, p < 0.001).

In the investigation of subsidence in relation to patient characteristics and demographics, men showed greater mean subsidence compared to women (1.48 vs. 1.16 mm, p < 0.001).

There was no difference between patients ≥ 75 and < 75 years of age regarding subsidence (1.29 vs. 1.30 mm, p = 0.674). No differences were found when comparing the subsidence of diabetic and non-diabetic patients (1.03 vs. 1.31 mm, p = 0.32). Similarly, nicotine consumption did not show an effect on subsidence (1.13 vs. 1.31 mm, p = 0.46). Alcohol consumption also showed no significant impact on subsidence (1.40 vs. 1.29 mm, p = 0.48).

A stem position without lateral bone contact showed a slightly greater, non-significant mean subsidence compared to those with lateral bone contact (1.40 vs. 1.20 mm, p = 0.029).

A significant difference was found between patients with a subsidence > 3 and ≤ 3 mm regarding revision surgery (5 vs. 5 patients, p < 0.001).

No significant association between revision surgery and smoking (p = 1.0), diabetes (p = 1.0), or alcohol abuse (p = 1.0) were found.

The calculated intra-observer reliability was 0,87 and the inter-observer reliability was calculated at 0,76.

## Discussion

The aim of this study was to analyze the migration pattern of a short stem by using an alternative radiological evaluation method, that could help surgeons and rehabilitation physicians to detect subsidence that may result in a change of postoperative treatment and counsel patients regarding early FWB. Outcomes focused on early postoperative implant migration, with subsidence measured using the SID. Results indicated that 6% (60 stems) demonstrated significant subsidence (SID > 3 mm), with a mean subsidence of 1.3 mm SID across the cohort. Complications resulted in the removal and exchange of 1% (10 implants) of the stems. Statistical analysis revealed no significant difference in subsidence related to diabetes, nicotine, alcohol consumption, high age, BMI or the stems lateral bone contact, but a significant difference in gender as well as weight.

In this study limitations lie in its retrospective nature and the short-term follow-up period, which may not fully represent long-term implant performance. The study only focuses on a single implant type which may limit the quality of the results due to missing of an ability to generalize. While the study design resulted in a randomization, there is still a risk of selection bias that reduces generalizability of our findings. Measurement limitations are present, as the Subsidence Index depends on standardized imaging.

The SID is a simple measurement method in everyday practice designed to offer an alternative to more elaborate and invasive techniques like RSA or EBRA for assessing implant subsidence in THA. The SID aims to quantify the migration of the femoral component within the femur by measuring the average movement along four distinct vectors. The advantage of the SID method is that it only requires conventional anterior–posterior radiographs, making its use in clinical settings easier without the need for specialized software. The measuring system can be used in an everyday setting and can detect patients with significant subsidence without further exposure to radiation. The measuring system can potentially be modified to any short stem by using any distinct marks on the short stem (eg. the tip of the short stem). Additionally, the analyzed intra- and inter-observer reliability indicates a good level of agreement between measurements with the SID.

While RSA and EBRA are known for their precision in detecting micromotions of implants, they are also known for their technical complexity and resource intensity.

RSA and EBRA are primarily used in research settings where these resources are available, and on a small number of patients [5, 8–10].

While the SID provides several advantages in terms of simplicity and accessibility, it has disadvantages regarding sensitivity and three-dimensional accuracy. Without the capacity for three-dimensional analysis, SID may overlook subtle but clinically significant movements, particularly in the rotational plane as well as varus and valgus changes. While acknowledging that varus, and especially valgus alignment, can have a significant effect on axial subsidence, Kutzner et al. showed in their study with 216 THAs using EBRA that it did not affect the clinical outcome [13].

The use of the SID in clinical practice also requires consistent standardized radiographs, which can be challenging in different hospitals.

Further research and comparative studies are planned and needed to potentially establish the SIDs efficacy relative to RSA and EBRA in clinical scenarios.

The average subsidence of the analyzed short stem was 1.3 mm SID with a range from 0 to 16.25 mm SID.

Studies indicate that most cementless short stems exhibit a subsidence between 0.40 to 1.5 mm measured with RSA or EBRA [7, 8, 10, 14]. This demonstrates that the short stem is comparable to other short stems in terms of subsidence.

Due to the retrospective design of this study the follow-up time regarding subsidence is significantly lower in this study. But it is important to acknowledge that a common characteristic of short stems is a minimal amount of migration before complete osteointegration [10, 14]. However, it is crucial that the short stem does not settle substantially, as this could increase the likelihood of aseptic loosening [6]. One study that analyzed the predictive value of early migration regarding aseptic loosening in 158 cementless short stems found that 2.7 mm within 24 months measured with EBRA-FCA was a relevant threshold [6]. This study used a cut-off value of SID 3 mm as it was deemed clinically relevant and is the next longer femoral head and is similar to the value calculated by Streit et al. [6].

The study found that patient characteristics such as diabetes status, nicotine, and alcohol consumption did not influence the severity of subsidence while weight had an influence on the severity of subsidence in this study population.

It has been previously shown that aseptic loosening, implant failure, periprosthetic infection and therefore also revision risk is influenced by the patient’s body weight [15–20]. One systematic review that investigated patient factors that influenced the outcome of the THA underlines that increased weight is associated with poor outcome [18].

The results regarding the influence of weight on subsidence found in this study further supports other studies on short stem subsidence– One study observed a clear and significant effect of absolute body weight on subsidence analyzing 202 short stems with EBRA-FCA after 2 years [9]. BMI on the other hand showed no influence on stem migration [9]. While this study found that absolute body weight above 100 kg was associated with greater subsidence, BMI did not show a significant influence either [9, 21, 22]. It appears plausible that a high absolute load on the stem lead to increased loading of the stem favoring subsidence. However, future studies using more elaborate measurement methods should reevaluate this particular group of patients.

The results indicate that the male gender significantly influences the subsidence rates.

Kutzner et al. as well as Jacobs et al. could also find these results with a slightly higher subsidence rate in men [9, 23]. Kutzner et al. describes different possible reasons for this correlation being either higher activity levels in men or increased weight [9].

In this study there was no significant negative effect on the subsidence by the short stems missing radiological contact to the lateral cortical bone. But there is a tendency that the subsidence was slightly higher with no contact seen in the radiograph.

Suksathien et al. found different results while analyzing 274 short stems [24]. Suksathien et al. recommends partial weight bearing when insufficient contact is seen in the post-operative radiograph as well as special attention creating sufficient contact intraoperatively because missing contact is a risk factor for higher subsidence [24]. Further and more extensive research is necessary to formulate scientifically proven recommendations, however partial weight bearing might be recommended if there are other risk factors present or if there is initial subsidence.

In conclusion**,** this study provides valuable insights into the subsidence behavior of a single design short stem. Overall, subsidence was relatively frequent while revision for this reason remained rare. While early full weight bearing appears safe, patients with risk factors present (men and increased body) and early subsidence should be counseled regarding this risk. Partial weight bearing until osseous integration might be an approach to mitigate this risk. Future studies should compare the SID to more elaborate, but resource intensive measuring tools like RSA or EBRA to underline the effectiveness of this method.
